# Emergency Department Utilization Due to Challenging Behavior in Children and Adolescents Diagnosed with Autism Spectrum Disorder

**DOI:** 10.3390/bs14080669

**Published:** 2024-08-02

**Authors:** Sara R. Jeglum, Alexandra Cicero, Jordan DeBrine, Cynthia P. Livingston

**Affiliations:** 1 Blank Children’s Hospital, Des Moines, IA 50309, USA; 2 Munroe-Meyer Institute, University of Nebraska Medical Center, Omaha, NE 68106, USA; 3 Kennedy Krieger Institute, Johns Hopkins University School of Medicine, Baltimore, MD 21205, USA

**Keywords:** autism spectrum disorder, emergency department, challenging behavior

## Abstract

Children and adolescents with autism spectrum disorder (ASD) are at a greater risk of seeking emergency department (ED) services during behavioral crises, such as acute aggression, suicidal or homicidal ideation, self-injury, or other types of challenging behavior (e.g., pica, dangerous behaviors). Research demonstrates children and adolescents with ASD often return to the ED due to challenging behavior, suggesting that gaps in care exist (e.g., follow-up appointments, referrals). However, the current knowledge basis is largely based on data from other countries. Given the unique landscape of healthcare in the United States, it is prudent to elucidate characteristics of children and adolescents with ASD who are seeking emergency care due to challenging behavior, as well as systems-level factors that both contribute to our understanding of challenging behavior and ASD in ED settings. In this study, we focus on frequency and characteristics of children and adolescents with ASD presenting to the ED with challenging behavior over the course of a 6-year period in the Midwest region of the United States. Clinical implications for ED staff are discussed.

## 1. Introduction

Individuals diagnosed with autism spectrum disorder (ASD) exhibit social and communication deficits and engage in restricted and repetitive behavior [1]. Moreover, challenging behavior (e.g., aggression, opposition) occurs in around 50% of children with intellectual and developmental disabilities (IDD), including ASD, with 10% of these cases considered to be severe challenging behavior [2]. Challenging behavior can include self-injury (e.g., self-biting, self-hitting, eye gouging), aggression towards others, and disrupting items. There are several antecedents that may result in individuals engaging in these topographies of behavior, including being denied access to tangible items, escape from demands, access to attention, and automatic reinforcement [2,3,4]. Individuals who are unable to communicate may engage in challenging behavior to communicate. In some cases, severe challenging behavior becomes unmanageable by caregivers at home or in community settings and results in an emergency department (ED) admission [5]. This may account for or contribute to more in-patient admissions for individuals diagnosed with ASD than their typically developing peers [6]. Unfortunately, healthcare workers may lack the skills and confidence to effectively work with individuals with ASD [7,8]. Patients with ASD may enter the ED engaging in challenging behavior, resulting in doctors treating the challenging behavior as a symptom of ASD and not as a symptom of an underlying medical condition [9]. This can result in individuals not receiving an accurate diagnosis or treatment. Many caregivers will bring their child to the ED due to challenging behavior, such as aggression resulting in an unsafe home environment for the child and other family members [10,11]. Caregivers may also bring their child to the ED who have feeding issues that cause the child to be malnourished and underweight [12]. Additionally, children diagnosed with ASD may engage in self-injury causing tissue damage and bruising to themselves [8]. Unfortunately, families may be unable to receive quality care due to waitlists or may be denied services due to the intensity of the challenging behavior [8]. Despite challenging behavior having communicative properties [13], evaluating functional variables of challenging behavior (e.g., pain, social contingencies) is not commonly taught in ED settings.

In ED settings, Efron et al. [14] found that physical restraints and medications were employed in 21% and 19% of cases, for patients diagnosed with ASD or IDD, respectively Additionally, Efron et al. [14] found one in four individuals who engage in challenging behavior, such as aggression and self-injury, were readmitted to the ED within three months of the initial admission due to the same concern. Less than 30% of patients admitted to the ED diagnosed with ASD were referred to a health or social service [14]. Therefore, it is possible that readmittance is high due to lack of resources provided to the families following their ED visit, such as follow-up appointments, support plans, and referrals [8,14].

Previous research has analyzed data from ED admissions of individuals diagnosed with ASD to identify potential patterns related to reason for admission, challenging behavior observed in the ED, and treatment, among other variables. For example, in 2021, Schott and colleagues [15] found self-harm and suicide were common reasons as to why individuals with ASD and attention deficit hyperactivity disorder (ADHD) were brought to the emergency department. Tint et al. [8] found that 54% of individuals diagnosed with developmental disabilities, including ASD, visited the ED due to aggression, 23% due to a medical reason, 13% due to self-harm or suicidal behavior, and 10% due to other reasons. Furthermore, they found restraints were used during 53% of psychiatric or behavioral visits. Crisis teams or psychiatrists were involved 56.7% of the time. These data suggest challenging behavior is a major contributing factor to ED visits for individuals with ASD [8].

Once in the ED, individuals with ASD typically encounter overstimulating environments, long waits, and difficulties obtaining referrals to potentially necessary services [14]. Research aimed at identifying similar patterns across ED visits for this population has allowed for modifications to EDs and subsequent analyses to detect the influence of these modifications made for this population. For example, Sadatsafavi et al. [16] conducted a review to identify different non-pharmaceutical interventions or modifications that can be implemented to reduce sensory challenges for individuals diagnosed with ASD when they are admitted to the ED. Suggested interventions included environmental modifications to decrease stimulation within the environment. For example, reducing wait time for patients, dimming lights, and keeping the same personnel with the patient throughout their admission [8,14,16] is acceptable.

Previous research in the literature has focused on small samples of individuals with ASD in the ED, largely in other countries (e.g., Melbourne, AUS, [14]; Ontario, Canada, [8]). Efron et al. [14] implemented a one-year retrospective review of patients diagnosed with ASD or IDD who engaged in challenging behavior. They found 84 patients were admitted to the ED with ASD or IDD engaged in challenging behavior. However, there was a total of 124 presentations, indicating some patients were admitted to the ED multiple times. Tint et al. [8] analyzed crisis reports that occurred during a two-year period for individuals diagnosed with developmental disabilities. Aggression was found to be the most common reason for individuals with developmental disabilities to be admitted to the ED (54%). Other reasons for admittance include medical emergencies, self-harm, or other non-specified problems. Few large-scale studies exist that focus on children with ASD presenting to the ED, specifically within the United States. Additionally, it is difficult to collect information that allows individual and system-level analyses (e.g., disposition, primary diagnosis, recidivism) due to limited access to patient health records.

Therefore, the aim of this study was to examine data of patients entering the ED diagnosed with ASD in three Midwestern states (Iowa, Illinois, and Wisconsin), to assess various characteristics of the ED encounter (e.g., reason for visit, disposition, recidivism), and discuss ways to reduce future visits for these patients.

## 2. Method

We conducted a six-year retrospective medical record review using data from Epic Health Record system, an electronic medical record software program for managing protected health information. We extracted data from EDs using Epic across nonprofit hospitals located in three Midwestern states (Iowa, Illinois, and Wisconsin) to identify the occurrence of ED visits amongst individuals diagnosed with ASD. Medical records were obtained from a total of 20 hospitals. Next, we analyzed demographic and clinical characteristics across patients to determine the degree to which challenging behavior influenced the patient’s admission to the ED.

### 2.1. Search Strategy

Inclusion criteria included (a) patients were between the ages of 2 and 22 at the time of the ED visit, (b) had at least one ED visit in which autism spectrum disorder was the primary diagnosis, and (c) the ED visit(s) occurred over a six-year period. Any patient who did not meet these criteria was excluded.

We first conducted a retrospective medical chart review in Epic of patients ages 2–22 with a primary diagnosis of ASD who presented to the ED between September 2017 and September 2023 in the Epic electronic healthcare record system for a Midwest hospital enterprise spanning three states (Iowa, Illinois, and Wisconsin). We used Slicer Dicer, a data extraction tool in Epic to conduct a customizable search across patient characteristics [17]. To collect data from patients with a diagnosis of ASD, we included search terms related to ASD (see Table 1). Terms queried in Epic included autistic disorder, pervasive developmental disorder, history of autism spectrum disorder, and Asperger syndrome. Table 1 includes a full list of diagnoses search terms. We performed further screening on included patients to identify additional ED visits that were excluded in the initial search due to the patient’s primary diagnosis not being listed as ASD.

Following the initial search in Slicer Dicer, we extracted and coded demographic and clinical characteristics for each ED visit. Specifically, we coded (a) the patient’s arrival time, (b) age, (c) sex, (d) primary diagnosis (categories included autism spectrum disorder, other developmental disabilities, mood disorder, behavioral diagnosis, mental health diagnosis, medical diagnosis, and other), (e) ED disposition (i.e., discharge, admit, transfer to another facility), (f) presence or absence of challenging behavior, and (g) the topography of challenging behavior. We included all visits to the ED for each patient who was included in the initial search using Slicer Dicer, including visits for which autism spectrum disorder was not the primary diagnosis in the ED, but the patient had the established history by chart review and the initial diagnostic information gathered using Slicer Dicer. Topographies of challenging behavior included aggression (e.g., hitting, punching, biting, kicking, pushing), self-injury (e.g., hand-to-head hitting, hand-to-body hitting, head banging), disruptive behavior (e.g., breaking items, throwing items, swiping off surfaces, hitting walls), suicidal ideation (i.e., verbalizing thoughts of harming self with or without intent), homicidal ideation (i.e., verbalizing thoughts of harming others with or without intent), and other (e.g., pica or ingesting inedible objects, elopement, dangerous acts such as attempts to touch electrical sockets or property destruction such as shattering windows). Primary diagnosis was taken directly from the ED visit information extracted from Epic. Autism spectrum disorder included all search terms from Table 1. Other developmental disabilities included intellectual disability global developmental delay, and attention deficit hyperactivity disorder. Mood disorder included disruptive mood dysregulation disorder, other specified or unspecified mood disorder. Behavioral diagnosis included aggression, self-injury, violent outburst, and other behaviorally based diagnoses (i.e., any diagnosis that is observation-based). Mental health diagnosis included depression and anxiety disorders, bipolar disorder, suicidal ideation, homicidal ideation, adjustment disorder, psychiatric problems, psychosis, schizophrenia, and mental-health-related complaints. Medical diagnosis included laceration, concussion, contusion, abrasion, constipation, foreign body in nose or stomach, generalized abdominal pain, infection, and other medically based diagnoses. Others included those that could not be categorized (e.g., social stress, family stress). No patient medical records were excluded; all records that met the inclusion criteria were screened and analyzed.

### 2.2. Interobserver Agreement

Interobserver agreement was completed for specific challenging behavior topographies (i.e., aggression, self-injury, disruptive behavior, suicidal and/or homicidal ideation, or other). Reliability data were collected on 26.4% of cases, and interobserver agreement was calculated using exact agreement methods. An agreement was scored if both observers coded that a topography was present. Exact interval agreement was scored for each topography category (i.e., self-injury, aggression, disruptive behavior, suicidal ideation, homicidal ideation, and other). Interobserver agreement averaged 86% (range, 80–100%).

## 3. Results

There were 193 individuals who met inclusion criteria, including 153 males (79.3%) and 40 females (20.7%). The median age was 15 years (range, 3 to 22 years). In total, there were 1325 ED visits, with 1001 (75.5%) of those visits related to challenging behavior, and 324 visits (24.5%) related to other issues (e.g., constipation, fever, seizure, vomiting, medication refill). Of the 193 individuals who visited the ED, 190 (98.4%) had challenging behavior, and 3 (2.6%) did not have challenging behavior. Primary diagnoses and challenging behavior characteristics are summarized in Table 2. ASD or a related diagnosis (e.g., Asperger syndrome) were reported as the primary diagnosis for 21.6% of total visits. Other primary diagnoses included a behavioral diagnosis (e.g., aggression, self-injury; 24.1%), mood disorders (9.8%), and mental health concerns (e.g., anxiety, depression, suicidal or homicidal ideation; 12.9%). Medical diagnoses were also reported when challenging behavior resulted in injury (e.g., concussion, laceration) in 29.5% of the sample.

Multiple topographies were often indicated during ED visits related to challenging behavior. One topography of challenging behavior was reported in 60.6% of visits, and more than one topography of challenging behavior was reported in 39.4% of visits. Challenging behavior that occasioned ED visits included aggression, which occurred in 658 visits (49.7% of all visits; 45.6% of total observed topographies). Suicidal and/or homicidal ideation, self-injury, disruptive behavior, and other behaviors (e.g., pica, elopement) occurred in 273 visits (20.6% of all visits; 18.9% of total observed topographies), 145 visits (10.9% of visits; 10.1% of topographies), 141 visits (10.6% of visits; 9.8% of topographies), and 225 visits (16.9% of visits; 15.6% of topographies), respectively.

Number of visits and ED dispositions are summarized in Table 3. A total of 53 individuals (27.5%) had one ED visit, and 140 individuals (72.5%) had more than one ED visit related to challenging behavior. Individuals had a median of 3 visits (range, 1 to 31) related to challenging behavior. Individuals were discharged from the ED 83.8% of the time, admitted 14.0% of the time, and transferred to a different facility 2.2% of the time.

### Challenging Behavior Visits Based on Age

Table 4 illustrates the differences in challenging behavior visits to the ED based on age within our sample. The visits were sorted by age to determine the number of individuals, number of visits, and topographies based on age ranges. The vast majority of the sample were between the ages of 13 and 18 years (n = 110; 49.9% of challenging behavior visits), with 11% of these visits being completed by those who were 18 years old at the time. There was an exponential increase in number of visits between ages two to six years and seven to twelve years of age. Notably, the age range of 19 to 22 had a smaller number of individuals and visits found. However, as it is a smaller age range, it cannot accurately be compared to the other age ranges. Previous research suggests that adults with ASD and developmental disabilities are more prevalent in ED visits compared to children [9]. A total of 20 of the 110 individuals in the 13 to 18 year age range (18.2%) were 18 years old at the time of their ED visit(s). It is likely that if the range were extended to a comparable level (i.e., 19 to 25) then a higher visit rate would be reflected.

## 4. Discussion

This is one of the first studies to investigate the unique presentation needs of children and adolescents diagnosed with ASD in the ED setting in three Midwestern states (i.e., Iowa, Illinois, and Wisconsin). Overall, our results suggest the majority of patients diagnosed with ASD presented to the ED due to concerns related to challenging behavior (75.5% of visits). Further, most patients were reported to engage in aggression (49.7% of visits) compared to other topographies, such as suicidal or homicidal ideation (20.6%), and self-injury (10.9%). Challenging behavior may emerge for a variety of reasons, including for operant reasons (e.g., social reinforcement, automatic reinforcement, lack of communication skills), underlying medical reasons that create an establishing operation for challenging behavior, or a combination of these and other factors. It is unclear if the large disproportion of ED visits due to challenging behavior is because of lack of specialized outpatient services, difficulties accessing primary health providers [18], limited psychiatric evaluations [19], or a combination of factors. Nonetheless, these findings demonstrate the need for adequate preventative and reactionary responses to adolescents and children with ASD experiencing behavioral crises in the ED.

Often, individuals with ASD may communicate pain through challenging behavior [9].

Therefore, ED providers should consider underlying medical conditions that may elicit challenging behavior. Providers are cautioned to avoid assuming challenging behavior is occurring solely based on an autism diagnosis, and instead focus on what the individual may be communicating by engaging in challenging behavior. For example, in the current study, a patient who was nonvocal and exhibited significant aggression was found to have a brain tumor, and upon its removal, ED visits ceased. In a similar example, a nonvocal 8-year-old boy engaged in pica (i.e., ingesting something inedible) of an unknown object, and an X-ray revealed radiopaque spots (i.e., areas that appear opaque on an X-ray), requiring surgery. In many individuals of the sample with limited communication skills, constipation was a primary concern of the ED visit; often, caregivers reported that aggression was common when their loved ones were constipated or experiencing gastrointestinal distress. A complete medical workup is essential to rule out underlying conditions causing challenging behavior [9]. Further, ED staff would benefit from acute training focused on ASD, challenging behavior, and crisis management, with a focus on managing and reducing aggression during a medical or behavioral crisis.

Many of the ED encounters included other mental and behavioral health conditions as the primary diagnosis (e.g., mood disorder), including for patients with an underlying ASD diagnosis requiring substantial to very substantial support. While comorbid mood disorders are common in ASD [15], appropriate identification of the visit concern is necessary to successfully produce adequate referrals. Additionally, challenging behavior is not a core symptom of ASD [1]. Nevertheless, the majority of those with a primary diagnosis of ASD during ED visits presented due to challenging behavior. This suggests a potential bias in providing services to individuals with ASD who present with challenging behavior, and that there is inadequate access to necessary behavioral services for those with ASD who also engage in challenging behavior. Service deserts and lack of access to well-trained providers represent a significant concern in managing care [20], particularly in rural areas [21]. Children in rural areas have less access to regular and specialty medical and mental health care and may be more likely to present to the ED [22,23]. Most individuals in our sample sought ED care more than once (73.1%), with a median of 3 visits, and an average of 5.3 visits (range, 1 to 31). Multiple ED visits suggest families are unable to access appropriate services to reduce challenging behavior. Given that an extensive body of research in the literature has documented the successful use of behavioral interventions derived from the principles of applied behavior analysis to reduce challenging behavior [2], providing access to behavioral services and parent training may reduce the probability of ED visits. However, future work is needed in this area to draw conclusions about the relationship between service access and ED visit frequency. Nevertheless, establishing equitable access to behavioral health centers and specialized treatment clinics in rural areas to reduce the prevalence of ED visits for children diagnosed with ASD is imperative.

Our findings support the expansion of patient and family-centered care in the ED. Nicholas and colleagues [24] found staff knowledge, training, and experience in ASD and attention to parent expertise were valued and beneficial to care. Caregivers of children with ASD are likely to have the greatest understanding of their child’s challenging behavior. Therefore, clinicians and ED staff should consult caregivers and identify common strategies that may reduce any imminent risk of harm related to challenging behavior. Moreover, clinicians and ED staff should emphasize a family-centered approach to treatment. For example, EDs could consider having a separate waiting area with adjustable lighting, available noise-cancelling headphones, wait-time strategies (e.g., tablets, toy area, visual cues), as well as availability of protective equipment (e.g., portable mats, arm guards) for both patients and staff [16].

This study is not without limitations. This is a sample of the United States, and not representative of the entire ED landscape for individuals with ASD. Notably, emergency department visits across the 20 total hospitals included in this study had an average of 52,800 visits per year across all ages. Additionally, our data collection involved identification based on the primary diagnosis of ASD during ED visits; therefore, the sample is likely an underrepresentation. This is especially true given ED providers do not consistently include ASD in the patient’s diagnosis list, even if ASD symptoms prompted the visit and underlying medical conditions were ruled out. Finally, our sample focused specifically on children and adolescents with a diagnosis of ASD.

Thus, future researchers could consider conducting a similar analysis across a larger sample size amongst heavily populated cities or other rural areas across the country. Additionally, given that adults with ASD have been found to visit the ED more frequently than children with ASD [9], an analysis to replicate and extend these findings to adults is warranted. We found that 27% of our sample were ages 18 and over. However, we limited our search to those up to the age of 22 years, and, thus, our ability to interpret these findings to adults is limited. It would be beneficial to compare child and adolescent visits to adult visits, and particularly to consider biological factors in aging, such as puberty, in the manifestation of challenging behavior.

Finally, the majority of patients in this study presented to the ED due to challenging behavior; future researchers may consider identifying common interventions used by healthcare providers in emergency settings to safely and effectively assess, manage, and support adolescents with ASD. Previous research has reported on the use of physical or chemical restraint [14] during ED visits for the patient’s and others’ safety. However, decision-making processes for using these restrictive procedures in EDs are not well described in the literature. Therefore, another area of future research is to evaluate the use of physical and chemical restraints, as these data were not available for the current study. A particular area of research to explore is the use of physical and chemical restraints following the implementation of a crisis prevention program. For example, Bernstein et al. [7] delineated a comprehensive crisis intervention program for healthcare settings, including emergency departments. Future work could evaluate long-term outcomes to reduce the utilization of physical restraints and medications, as well as staff injuries [6].

Our findings suggest children and adolescents with ASD are at a greater risk of presenting to the ED due to challenging behavior [5,8]. Preventative healthcare and increasing access to services for the assessment and treatment of challenging behavior should be considered a public health issue, particularly in rural areas where resources may be scarce. Nevertheless, increasing awareness of this complex issue can lead to improved care for individuals and their families, thereby decreasing emotional, physical, and financial stress.

## Figures and Tables

**Table 1:** List of patient diagnoses search terms.

Search Terms
Autism
Autism disorder
Autism spectrum
Autistic behavior
Autism spectrum disorder
Autistic disorder
Autistic spectrum disorder
Residual autism spectrum requiring support with accompanying intellectual impairment
Autism spectrum disorder associated with neurodevelopmental, mental, or behavioral disorder requiring support (level 1)
Autism spectrum disorder with accompanying intellectual disability without language impairment requiring support (level 2)
Autism spectrum disorder with accompanying intellectual impairment requiring very substantial support (level 3)
Autism spectrum disorder requiring support associated with another neurodevelopmental, mental, or behavioral disorder
Autism spectrum disorder with accompanying intellectual disability without language impairment requiring substantial support
Autism spectrum disorder with accompanying language impairment without intellectual disability requiring substantial support
Autism spectrum disorder requiring very substantial support with accompanying language impairment with accompanying intellectual disability
History of autism
Active autistic disorder
History of autism spectrum disorder
Asperger syndrome
Asperger’s disorder
Asperger’s syndrome
Pervasive developmental disorder
Pervasive developmental disorder, active
Other specified pervasive developmental disorders, current or active state
Pervasive developmental disorder (PDD)
Pervasive developmental disorder (PDD), active

**Table 2:** Primary visit diagnoses and challenging behavior characteristics.

Diagnoses and Challenging Behavior Characteristics		
*Primary Diagnosis*	*Number*	*Percentage*
Autism spectrum disorder	287	21.7%
Other developmental disabilities	16	1.2%
Mood disorder	130	9.8%
Behavioral diagnosis	328	24.8%
Mental health diagnosis	158	11.9%
Medical diagnosis	395	29.8%
Other	11	0.8%
*Challenging Behavior Topographies*	*Number*	*Percentage*
Self-injury	145	10.1%
Aggression	655	45.6%
Disruptive behavior	141	9.8%
Suicidal and/or homicidal ideation	271	18.9%
Other	223	15.5%

**Table 3:** Number (n) of visits and ED dispositions.

Visit Characteristics			
*Number of Visits for Individuals*	*Challenging Behavior Visits by Individual*	*Non-Challenging Behavior Visits by Individual*	*Total Number of Visits*
1	53	44	97
2	24	25	98
3	23	15	114
4	20	12	128
5	9	9	90
6 or more	61	11	798
*Disposition*	*Challenging Behavior*	*Non-Challenging Behavior*	*Total*
Discharge	n = 787; 78.6%	n = 323; 99.9%	n = 1110; 83.8%
Admit	n = 185; 18.5%	n = 1; 0.1%	n = 186; 14.0%
Transfer to Another Facility	n = 29; 3.0%	n = 0	n = 29; 2.2%

**Table 4:** Emergency department utilization due to challenging behavior based on age.

Category	Data							
*Age Range*	*Number of Individuals*		*Number of Visits*		*Visits Median and Range*		*Percentage of Visits*	
2 to 6 years	12		17		M = 1; range, 1–4		1.7%	
7 to 12 years	75		289		M = 3; range, 1–24		28.9%	
13 to 18 years	110		499		M = 3; range, 1–27		49.9%	
19 to 22 years	32		196		M = 4; range, 1–21		19.6%	
Total	190 *		1001		M = 3; range, 1–31		100%	
*Challenging Behavior Topographies*	*2 to 6 Years*		*7 to 12 Years*		*13 to 18 Years*		*19 to 22 Years*	
Self-injury	3	10.7%	61	13.7%	61	8.7%	20	7.7%
Aggression	15	53.6%	206	46.3%	314	44.7%	120	46.2%
Disruptive behavior	2	7.1%	45	10.1%	66	9.4%	28	10.8%
Suicidal and/or homicidal ideation	3	10.7%	60	13.5%	154	21.9%	54	20.8%
Other	5	17.9%	73	16.4%	107	15.2%	38	14.6%

Note. * Thirty-nine individuals were seen multiple times across age groups.

## Data Availability

The original contributions presented in the study are included in the article, further inquiries can be directed to the corresponding author/s.
